# Effect of Exercise on the Cognitive Function of Older Patients With Type 2 Diabetes Mellitus: A Systematic Review and Meta-Analysis

**DOI:** 10.3389/fnhum.2022.876935

**Published:** 2022-04-28

**Authors:** Yi-Hui Cai, Zi Wang, Le-Yi Feng, Guo-Xin Ni

**Affiliations:** ^1^School of Sports Medicine and Rehabilitation, Beijing Sport University, Beijing, China; ^2^School of Sport Science, Beijing Sport University, Beijing, China

**Keywords:** exercise, type 2 diabetes mellitus, older adults, cognition function, meta-analysis

## Abstract

**Background:**

Aging and type 2 diabetes mellitus (T2DM) are important risk factors for the development of cognitive deterioration and dementia. The objective of this research was to investigate the effects of an exercise intervention on cognitive function in older T2DM patients.

**Methods:**

Eight literature databases (PubMed, EBSCO, Scopus, Embase, The Cochrane Library, Web of Science, Ovid, and ProQuest) were searched from inception to 20 January 2022. The researchers examined randomized controlled trials (RCTs) that evaluated the impact of exercise on the cognitive performance of older T2DM patients. The Cochrane risk-of-bias tool (ROB 2) for RCTs was used to assess each study. The quality of evidence was assessed using the GRADE (grading of recommendations, assessment, development, and evaluations) approach. The mini-mental state examination (MMSE), Modified MMSE (3MSE), and Montreal cognitive assessment (MoCA) were used to evaluate the cognitive outcomes. We performed a subgroup analysis with stratification according to exercise intervention modality, duration, and cognitive impairment.

**Results:**

Five trials were eligible, with a total of 738 T2DM patients. The combined findings revealed that exercise improved global cognitive function significantly (standardized mean difference: 1.34, 95% confidence interval: 0.23–2.44, *p* < 0.01). The effect of exercise on global cognitive performance was not significantly influenced by intervention modality, intervention duration, or cognitive impairment in the sub-group analysis (*p* > 0.05). In the studies that were included, no relevant adverse events were reported.

**Conclusion:**

Exercise is beneficial in improving global cognitive function in older adults with T2DM. Studies with bigger sample sizes and higher quality are additionally expected to draw more definite conclusions.

**Systematic Review Registration:**

[https://www.crd.york.ac.uk/PROSPERO/#recordDetails], identifier [CRD42022296049].

## Introduction

With changes in people’s lifestyles and the aging population, the prevalence of diabetes has been increasing and is expected to increase to 783.2 million by 2045, but is even higher in older age groups (Sun et al., 2021). Type 2 diabetes mellitus (T2DM) represents 90–95% of all diabetes cases (American Diabetes Association, 2021). In the elderly population, diabetes leads to the onset of multimorbidity, polypharmacy, and disability, thus increasing the economic burden on society and patients’ families (Salcedo Rocha et al., 2018). Global diabetes-related health spending is also estimated to increase from $966 billion in 2021 to $1,054 billion in 2045 (Sun et al., 2021).

Patients with T2DM are more likely to experience cognitive decline, which is more pronounced in older patients with T2DM, than those without the disease (Kotsani et al., 2018). However, cognitive dysfunction is often easily overlooked in patients with T2DM (Liu et al., 2020). Long-term chronic hyperglycemia can impair brain function and cause peripheral vascular complications such as neuropathy, stroke, and white matter lesions, which lead to cognitive dysfunction in patients with T2DM (Alosco et al., 2012; Luchsinger, 2012; Ho et al., 2013; Koekkoek et al., 2015). Cognitive impairment and dementia lead to reduced adherence to proper treatment for T2DM, which results in increased risks of complications such as cognitive dysfunction (Althubaity et al., 2021). This suggests that in patients with T2DM, the risk of cognitive deterioration can be reduced through well-controlled blood glucose levels (West et al., 2014).

Cognitive deterioration is more severe in older patients with T2DM and thus should be prevented or treated using effective measures. Exercise establishes a clinical treatment pathway for T2DM in primary care (Rehn et al., 2013; Rossen et al., 2015), and contributes to the reduction of the burden of chronic diseases and improvement of public health (Rossen et al., 2015). Many researchers have emphasized the relevance of physical activity for cognition, and the latest research suggests a positive association between exercise and all cognitive domains (Liu et al., 2021). Although some trials have shown exercise improves cognitive function in patients with T2DM, their results are inconsistent (Yanagawa et al., 2011; Espeland et al., 2017a; Chantre Leite et al., 2020; Molina-Sotomayor et al., 2020; Martínez-Velilla et al., 2021). One study found that 6 months of progressive aerobic and resistance training improved global and domain-specific cognitive function in patients with T2DM (Callisaya et al., 2017a). However, another study found that after 6 months of aerobic and resistance exercise with a lifestyle intervention, the cognitive function of patients with T2DM was negatively affected (Fiocco et al., 2013).

A meta-analysis of the influence of physical exercise on cognitive performance in diabetes patients that was published in 2021 uncovered that exercise improved cognitive function in patients. However, the interventions included in the study contained diet and exercise, not an exercise intervention alone, and the types of studies consisted of cohort studies and RCTs (Wang et al., 2021). Zhao et al. (2018) tried to systematically analyze the effects of physical exercise on cognitive function in people with T2DM, insulin resistance, or impaired glucose tolerance (IGT) in 2018. Their results indicated limited data supporting the idea that physical activity may enhance some cognitive functions in older adults with T2DM or IGT, but the effects were inconsistent. As the included studies include both observational and non-randomized controlled studies, further research is required. The question we wanted to investigate was whether exercise alone could improve cognitive function in older patients with type 2 diabetes.

The objective of this article was to systematically analyze the existing evidence from RCTs on the influence of regular physical activity on cognitive function in older T2DM patients. Thus this study reduces the uncertainty about the effects of exercise interventions on cognitive function in older patients with T2DM. It provides evidence for future non-pharmacological prevention and treatment methods for cognitive impairment in the older T2DM population.

## Materials and Methods

The analysis methodologies and eligibility criteria were set ahead of time and documented in a PROSPERO-registered protocol (CRD42022296049). The Preferred Reporting Items for Systematic Reviews and Meta-analyses were used in this meta-analysis (Page et al., 2021; Supplementary Material, PRISMA 2020 checklist).

### Eligibility Criteria

The following criteria were used to determine whether or not trials should be included in this review. The study comprised older T2DM patients (aged 60 years and more). Participants could be male or female and from any country. Any structured exercise training program was completed for at least 8 weeks without the use of any additional treatment approaches or lifestyle changes. The fitness plans might be done anywhere (e.g., laboratory, home, or gym). Patients who did not receive contact/usual care were not on the waiting list, did not undertake a sham exercise or passive training, or did not receive an alternative active treatment were all eligible for comparison. To ascertain the outcome, any validated cognitive function test conducted at baseline and follow-up after exposure to an exercise intervention can be used. RCTs completed in humans were considered eligible for inclusion in the study. Conference proceedings, guidelines, dissertations, commentaries, reviews, animal model studies, and letters were all eliminated from consideration. Articles for which there was no full text or raw data were also eliminated. Table 1 presents the PICOS criterion’s inclusion and exclusion criteria.

**TABLE 1:** The inclusion and exclusion criteria under the PICOS criteria.

Parameter	Defined criteria for the present study
P (participants)	Older patients with T2DM (aged ≥ 60 years)
I (intervention)	Structured exercise for at least 8 weeks
C (comparison)	Standard care, waiting list, sham exercise, passive training, or active therapy options
O (outcomes)	Cognitive function
S (study design)	Randomized controlled trials

### Search Strategy and Study Selection Processing

Two review raters conducted a thorough literature search to find relevant articles (Y-HC and ZW). The search period covered the years from inception (1818∼2004) to 20 January 2022. The following electronic databases were searched: PubMed, EBSCO, Scopus, Embase, The Cochrane Library, Web of Science, Ovid, and ProQuest. No language or publication status restriction was set. The search keywords included “participant” (e.g., “diabetes mellitus, type 2”), “intervention” (e.g., “exercise”), and “outcomes” (e.g., “cognition,” “executive,” “attention,” and “memory”). The description of the complete search strategy is provided in PDF (Supplementary Material, Database Search formula). We used the Mesh database for the PubMed search and combined Mesh terms with entry terms. After that, we made changes to other databases. Furthermore, the following filters were used: “randomized controlled trial” (publication kinds), “randomized” (title/abstract), and “placebo” (title/abstract). EndNote reference software (EndNote X9, Clarivate Analytics, Philadelphia, United States) was used to collate and save the trials, and duplicates were deleted. Two researchers (Y-HC and ZW) evaluated the titles and abstracts separately to select the papers that fit the criteria, and then read the entire texts to determine final eligibility. Until a consensus was reached, any disagreements amongst the study authors were handled through discussion or third-party consultation (L-YF or G-XN).

### Data Extraction

All of the data was extracted and compiled independently by two researchers (Y-HC and ZW). We gathered the following details: (1) trial characteristics (lead author, year of publication, trial aim, trial design, inclusion/exclusion criteria, sample size, and allocation method); (2) participant characteristics (diabetes diagnosis, age, sex, BMI, length of diabetes diagnosis, and medication); (3) intervention (type, duration, frequency, and intensity); and (4) outcome measurements (all relevant cognitive outcomes and measurement tools). In the evaluation of the intervention effects at various time points, only the value obtained at the latest time point was taken into account. We contacted the authors to collect the original data if there was no relevant data in the paper. When the two reviewers couldn’t agree, a third reviewer was brought in to help reach a conclusion (L-YF or G-XN).

### Quality and Risk-of-Bias Assessment

Individual articles were evaluated separately by the two reviewers (Y-HC and ZW) using the Cochrane risk-of-bias tool for randomized trials (ROB 2), in accordance with Higgins et al.’s recommendations (Higgins et al., 2020). The questions in ROB 2 analyze five domains: the randomization procedure, deviations from the planned treatments, missing outcomes, outcome measurement, and reporting results selection. “Yes (Y),” “probably yes (PY),” “no (N),” “probably no (PN),” or “no information (NI)” was used to respond to the questions. The ROB 2 algorithm categorized bias risk as “low,” “high,” or “some concerns” at the conclusion of each domain. ROB 2 produced an overall rating after assessing the five domains. Publication bias could not be assessed because fewer than 10 studies were included. In addition, the GRADE (Grades of Recommendation Assessment, Development, and Evaluation) procedures were employed to assess the evidence quality (Guyatt et al., 2011). The GRADE system is a strategy for evaluating evidence quality based on the risk of bias, indirectness, inconsistency, imprecision, and publication bias. Evidence is rated as high, moderate, low, and very low in terms of quality. Disagreements between reviewers were addressed by additional discussion or, if necessary, by the involvement of a third reviewer (L-YF or G-XN).

### Data Synthesis and Statistical Analysis

The meta-analysis was carried out using R Studio Version 4.1.2 software (RStudio, Boston, Massachusetts, United States), the R package used is “library(meta).” We gave a narrative overview of the results for outcomes that could not be aggregated. The standardized mean differences (SMDs) of pre- and post-interventions were computed and weighted by inverse variances, taking into account the various outcomes and units of cognitive measures utilized in the research. SMD, calculated as the mean difference (MD) divided by the standard deviation, was pooled in random-effects model in which weight of the study was determined by the D-L method (Borenstein et al., 2009). The SMD was Cohen’s *d*, small, moderate, and high effect sizes were represented by Cohen’s *d* values of 0.2, 0.5, and 0.8, respectively (Higgins and Green, 2011). Based on the assumption of varying genuine effect sizes (Higgins and Green, 2011), a random-effects model was utilized, which takes into account study variance and weighs each study appropriately. *I*^2^ statistics and the Cochran *Q* test were used to determine heterogeneity. Small, medium and large quantities of heterogeneity are indicated by *I*^2^ values of 25, 50, and 75%, respectively (Higgins and Green, 2011), or a *p*-value of ≥0.10 for the Q test (Higgins et al., 2003). Sensitivity analyses were carried out by removing studies one by one from the meta-analysis.

We pooled the total scores of the scales assessing global cognitive function (MMSE, 3MSE, and MoCA). The cognitive function of T2DM patients was assessed by the MMSE, 3MSE, and MoCA scales, and they were all high-merit indicators, with higher scores associated with better cognitive function. Due to the different scoring criteria, we used SMD for aggregation rather than MD. Some domain-specific cognitive functions, such as attention, executive function, and memory function, were not be aggregated. RCTs assessing the effects of exercise therapy on specific cognitive skills in older adults with T2DM were too few and inadequate to allow for a meta-analysis. Therefore, we did not perform aggregate analysis of these outcomes.

Subgroup analyses were conducted on the basis of categorical factors such as exercise intervention modality, intervention duration, and cognitive impairment to evaluate possible moderating effects. This study divided exercise intervention modalities into two categories: (Sun et al., 2021) single-mode exercise (aerobic or resistance exercise alone); and (American Diabetes Association, 2021) multimodal exercise (aerobic exercise, resistance training, functional exercise, flexibility training, and balance training. Moreover, intervention duration (≥12 or <12 months) and cognitive impairment (with or without cognitive impairment).

## Results

### Literature Search and Study Selection

**FIGURE 1:**
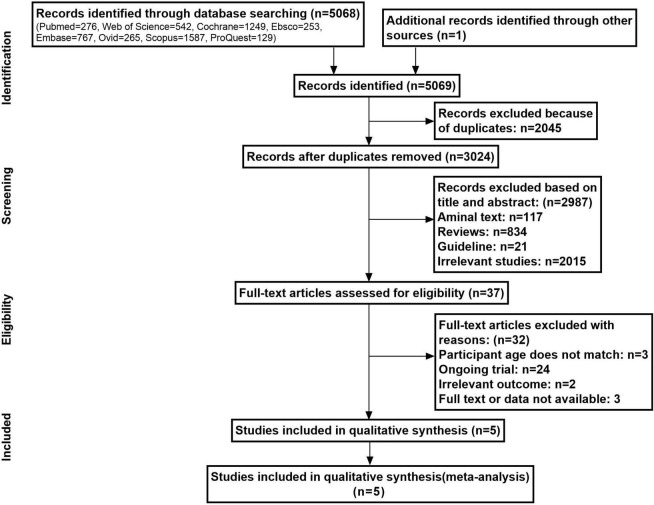
Flow diagram of the literature search and article selection.

The computerized database retrieval yielded a total of 5,069 entries. Figure 1 depicts the study selection flowchart. A total of 2,045 duplicate entries were removed, while 2,987 irrelevant data were eliminated based on their titles and abstracts. Following that, 37 full-text papers were reviewed for eligibility, with 32 being rejected. The following were some of the reasons: Subjects’ age does not match (*n* = 3), ongoing trial (*n* = 24); irrelevant outcome (*n* = 2); and no full text or data available (*n* = 3). Irrelevant outcome referred to outcome indicators for specific cognitive function domains, such as attention, executive function, and memory function.

### Characteristics of the Included Studies

Finally, the final analysis comprised five studies with a total of 738 individuals, three of which were from various countries (Zhu et al., 2015; Espeland et al., 2017a; Yamamoto et al., 2021) (the United States, China, and Japan, respectively) and two from Spain (Molina-Sotomayor et al., 2020; Martinez-Velilla et al., 2021). Table 2 lists the features of the studies that were included. The included literature was published from 2015 to 2021. The sample sizes for the research varied from 35 to 415 people. Two studies were conducted in aT2DM population without cognitive impairment (Espeland et al., 2017a; Yamamoto et al., 2021), two others on the T2DM population with cognitive impairment (Zhu et al., 2015; Molina-Sotomayor et al., 2020), and one study included T2DM populations with and without cognitive impairment (Martínez-Velilla et al., 2021). Among the included RCTs, four compared an exercise group with a non-exercise control group (Zhu et al., 2015; Molina-Sotomayor et al., 2020; Martínez-Velilla et al., 2021; Yamamoto et al., 2021) (e.g., maintained daily activities, education, and usual care), and only one compared exercise to gentle movements (Espeland et al., 2017a) (e.g., stretching and flexibility training). Two studies included aerobic activity, strength training, functional exercise, flexibility training, and balance training as part of a multimodal exercise design (Espeland et al., 2017a; Martínez-Velilla et al., 2021). Only aerobic (Zhu et al., 2015; Molina-Sotomayor et al., 2020) or resistance training were utilized in the remaining three trials (Yamamoto et al., 2021). The exercise intervention duration ranged from 3 to 24 months, and the exercise frequency varied from 3 to 7 sessions a week, lasting for 105–300 min/week. The duration of the exercise intervention protocols was <150 min/week in one study, <5 days/week in another study, and <12 months in two studies. Intervention modality, intervention duration, and cognitive impairment were all regarded as key confounding variables in the research. In none of the investigations, there were any adverse events linked to exercise.

**TABLE 2:** Characteristics of the included studies.

Author, year, country	Patients condition	Age range (years)	Sample (male/female)	Comparison	Intervention	Length (min/week)	Cognitive outcomes	Adverse event
Martínez-Velilla et al. (2021), Spain	T2DM	≥75	103 (50/53)	Usual care	Multimodal exercise, 40 min/day, 5–7 days/week for 3 months	200–280	Global cognition function/MMSE	None
Yamamoto et al. (2021), Japan	T2DM without cognitive impairment	70–79	35 (19/16)	Maintain daily activities	Bodyweight resistance and elastic band exercises, 15 min daily for 12 months	105	Global cognition function/MMSE	None
Molina-Sotomayor et al. (2020), Spain	T2DM with cognitive impairment	≥65	107 (0/107)	Maintained daily activities	Walking-based training, 60 min/day, 3 days/week for 6 months	180	Global cognition function/MMSE	None
Espeland et al. (2017a), United States	T2DM without cognitive impairment	70–89	415 (155/260)	Education workshops, stretching exercise, and flexibility training	Multimodal exercise, 50 min/day, 5–6 days/week for 24 months	250–300	Global cognition function/3MSE; processing speed/DSC-WAIS-III; memory function/HVLT-R; executive function/n-back task, TSP, EFT	None
Zhu et al. (2015), China	T2DM with cognitive impairment	≥60	78	Routine nursing	Baduanjin and routine nursing care, 40 min/day, 5 days/week for 12 months	200	Global cognition function/MoCA	None

*MMSE, mini-mental state examination; 3MSE, modified mini-mental state examination; MoCA, Montreal cognitive assessment; DSC-WAIS-III, Digit Symbol Coding Test-Wechsler Adult Intelligence Scale, Third Edition; HVLT-R, Hopkins verbal learning test-revised; TSP, task switching paradigm; EFT, Eriksen flanker task.*

### Risk-of-Bias Assessment

**FIGURE 2:**
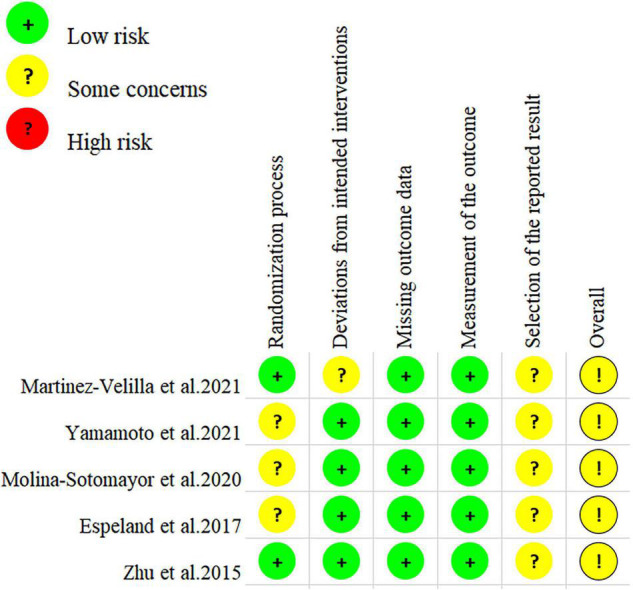
Risks of bias of the included study according to the Cochrane Collaboration guidelines.

Using the GRADE system, the included studies’ methodological quality was rated as very low. Figure 2 depicts the risk-of-bias plot and the author’s assessment of the risk-of-bias items. Regarding the first criterion, the allocation sequences were randomized in all the included studies, but three studies did not specify whether allocation concealment was used (Espeland et al., 2017a; Molina-Sotomayor et al., 2020; Yamamoto et al., 2021). There are only two included studies that specifically describe randomization methods for RCTs, such as the random number table method and computer-generated random numbers (Zhu et al., 2015; Martínez-Velilla et al., 2021). None of the studies had missing baseline characteristics. In the research by Martínez-Velilla et al. (2021), we identified participants who moved to another group in both groups for departures from the targeted treatments. In the missing outcome data criteria, there was no risk of bias. Assessor blinding was not found in three trials when it came to outcome measurements (Zhu et al., 2015; Molina-Sotomayor et al., 2020; Yamamoto et al., 2021). In all the studies, the pre-specified analysis plans did not describe the statistical analysis methods and might confer potential risks, so we assigned “some concerns” in these studies.

### Synthesis of the Results (Global Cognitive Function)

**FIGURE 3:**
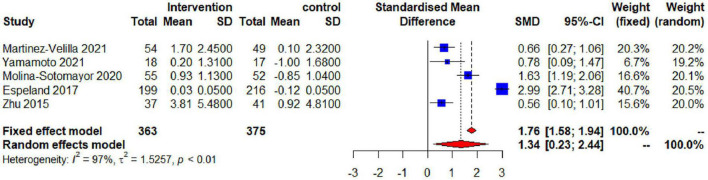
Effectiveness of exercise for improving global cognitive function in the elderly.

The impact of physical exercise on global cognitive function in elderly patients with T2DM is shown in Figure 3. The effects of physical exercise on overall cognitive performance were assessed using the MMSE, 3MSE, and MoCA scales in five studies with a total of 738 individuals. The combined analysis demonstrated that exercise therapies had a significant impact on improving global cognitive function. However, heterogeneity among the studies was discovered [SMD = 1.34, 95% confidence interval (CI): 0.23–2.44, *I*^2^ = 97%, *p* < 0.01]. The high heterogeneity of global cognitive function between research highlights the need of taking into account a variety of moderator variables when analyzing the effects of exercise.

### Subgroup Analysis

**FIGURE 4:**
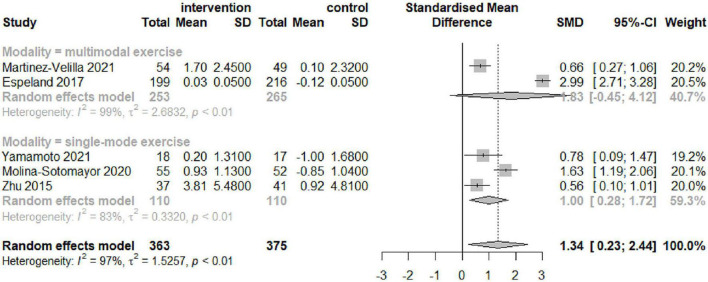
Forest plot of the results of the subgroup analysis for intervention modality (multimodal and single-mode exercises) as a moderator.

**FIGURE 5:**
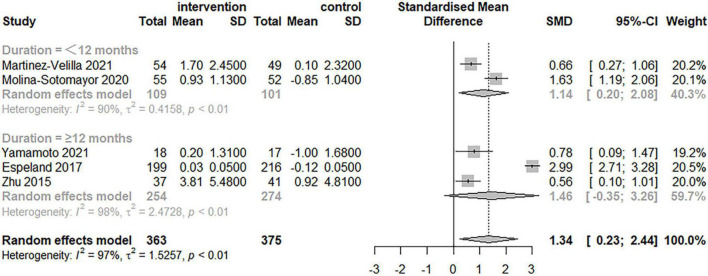
Forest plot of the results of the subgroup analysis for duration as a moderator.

**FIGURE 6:**
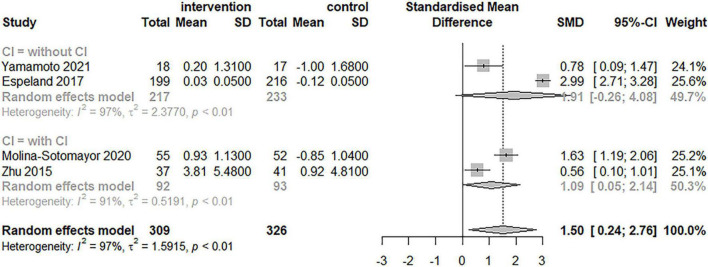
Forest plot of the results of the subgroup analysis for cognitive impairment as a moderator.

Table 3 and Figures 4–6 summarize the results of the subgroup analyses for global cognitive function. Subgroup analyses were conducted based on the result of the five trials, which included exercise intervention modality and duration, as well as cognitive impairment, following a meta-analysis for global cognitive function. The impact of exercise on global cognitive performance was not significantly moderated by intervention modality, duration, or cognitive impairment in subgroup analyses.

**TABLE 3:** Subgroup analysis for exercise and cognitive function.

Categorical moderator	Category	No. of studies	Cohen’s *d*	95%CI	*I*^2^%	Test of heterogeneity		
						*Q*	*d.f.*	*P* value
Modality	Multimodal exercise	2	1.83	−0.45 to 4.12	98.9%	0.47	1	0.49
	Single-mode exercise	3	1.00	0.28 to 1.72	82.9%			
Duration, month	≥12	3	1.46	−0.35 to 3.26	97.9%	0.09	1	0.76
	<12	2	1.14	0.20 to 2.08	90.1%			
Cognitive impairment	With cognitive impairment	2	1.09	0.05 to 2.14	90.9%	0.44	1	0.51
	Without cognitive impairment	2	1.91	−0.26 to 4.08	97%			

### Sensitivity Analysis

**FIGURE 7:**
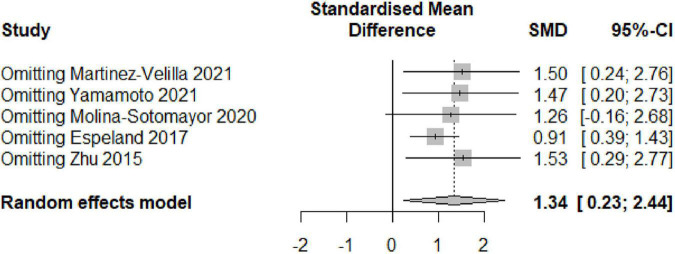
Sensitivity analysis based on the random-effects model.

To analyze the impact of each study on the overall findings, a sensitivity analysis was undertaken utilizing the sequential omission of individual studies. The findings of the sensitivity analysis did not vary significantly when any studies were excluded, indicating that the results of the pooled effect (SMD) of the exercise interventions on cognitive performance were generally stable. Figure 7 shows the results of the sensitivity analysis using the random-effects model.

## Discussion

This systematic review compiled evidence from recent RCTs and meta-analyzed the role of exercise on global cognitive function in older T2DM patients. The study demonstrates that exercise could significantly enhance global cognitive performance in older patients with T2DM. Unlike recently published studies (Zhao et al., 2018; Cooke et al., 2020; Wang et al., 2021), this study was an innovation in that the included studies were all RCTs that analyzed only the evidence of the impact of physical exercise on cognitive performance in older T2DM patients, controlling for some confounding factors of intervention modalities (e.g., diet, cognitive training, and lifestyle interventions) and populations.

Numerous studies have shown that exercise can promote neural regeneration and synaptogenesis in the hippocampus (Ma et al., 2017; De la Rosa et al., 2020), promote the release of neurotrophic factors (De la Rosa et al., 2020), reduce inflammatory processes (De la Rosa et al., 2020), and improve cognitive performance in different elderly populations. However, none of these recent meta-analyses systematically analyzed the impact of regular exercise on cognitive function in older patients with T2DM. Exercise’s effects on cognitive function in patients with T2DM have been studied (Yanagawa et al., 2011; Fiocco et al., 2013; Chantre Leite et al., 2020; Molina-Sotomayor et al., 2020; Leischik et al., 2021), but the results vary. This study summarized the results of previously published articles and exhibited that long-term exercise regularly can improve global cognitive function (MMSE, 3MSE, and MoCA) in older adults with T2DM, which may lower the risk of complications and enhance the quality of life of patients to some extent. In the trials that were included, no adverse effects connected to exercise were recorded. As a result, regular physical activity may be safe and effective for enhancing cognitive function in elderly patients with T2DM.

The neurophysiological mechanisms of exercise as an adjunctive treatment modality for patients with T2DM that modulates cognitive processes are complex. Increasing growth factors and neuroplasticity, inhibiting inflammatory marker production, improving vascular function, and modulating the hypothalamic-pituitary-adrenal axis are the bases for exercise-induced cognitive improvement (Quigley et al., 2020). From another perspective, exercise improves overall cognitive function by inducing the release of the brain-derived neurotrophic factor (Rasmussen et al., 2009; Enette et al., 2017) and insulin-like growth factor 1 (Cassilhas et al., 2007) to promote structural and connectivity changes in the hippocampus, temporal lobe, frontal lobe, and corpus callosum (Erickson et al., 2009; Gomez-Pinilla and Hillman, 2013; Voss et al., 2013; Maass et al., 2016), which improve certain cognitive functions such as executive function, attention, processing speed, and memory, among other domain-specific cognitive functions, in older adults. In addition, maintaining excellent long-term glycemic management with exercise may minimize the risk of cognitive impairment.

Our study showed that exercise significantly improved global cognitive function and effectively reduced the cognitive impairment status in elderly patients with T2DM. In these patients, improving cognitive functional status can improve their self-management ability and treatment compliance, effectively prevent the occurrence and exacerbation of complications, and to some extent, improve their general health condition. Exercise is a simple and easy-to-learn therapy choice for T2DM that may be practiced in the community or at home. Moreover, it is a low-risk, low-cost therapeutic option with several physical and psychological advantages applicable to both primary care and hospitals.

We summarized other specific cognitive function domains included in the study (as shown in Table 1), such as processing speed (DSC-WAIS-III), memory function (HVLT-R), and executive function (n-back task, TSP, and EFT) because of the evidence that exercise can improve cognitive function in specific areas of the brain, such as attention, executive function, and memory function in patients with T2DM (Baker et al., 2010; Callisaya et al., 2017b; Espeland et al., 2017b). Unfortunately, RCTs evaluating the effects of exercise therapies on particular cognitive skills in older persons with T2DM are scarce and insufficient, making meta-analyses impossible.

The results of the subgroup analysis performed in this study showed that confounding factors such as the type and duration of exercise intervention as well as the existence of cognitive impairment had not significantly affected the improvement of global cognitive function by exercise. However, the impact of an exercise intervention on global cognitive function exhibited high heterogeneity, according to our data. In the subgroup analysis based on exercise type, multimodal exercise interventions were not better than single-exercise modalities in improving global cognitive function in older T2DM patients. This matches the findings of two previous meta-analyses, which showed that both single-aerobic exercise and aerobic exercise paired with additional exercise therapies helped diabetes patients improve cognitive function (Wang et al., 2021). The other meta-analysis of the improvement of cognitive performance by exercise in older adults by exercise showed the benefits of both aerobic and resistance training on cognitive function (Northey et al., 2018). However, some studies have shown more cognitive benefits of multi-component exercise interventions such as aerobic, resistance, balance, and flexibility training in healthy older adults, especially when combining aerobic and resistance exercises (Colcombe and Kramer, 2003; Kramer and Colcombe, 2018). It has been shown that there is a significant positive correlation between balance function and cognitive function, and that older adults with poor balance are at greater risk for impaired cognitive function (Meunier et al., 2021). Cognitive decline in older adults can be prevented through exercise prescriptions that include balance training. In addition, regular aerobic walking training outdoors can improve cognitive function by allowing older adults to maintain greater balance in their bodies (Battaglia et al., 2020). All of these exercise modalities have been shown to improve cognitive function in older adults. There is some ambiguity in the choice of exercise intervention methods, which may be related to the specific populations, settings, and intervention duration of the different studies. Therefore, further research is needed in this area. In addition, there was disagreement about the duration of the intervention. In our research, intervention duration does not constitute a confounding factor affecting intervention outcomes. This is consistent with the results of another meta-analysis, where the total duration of the intervention and the weekly exercise intervention hours did not significantly affect cognitive function in older adults (Sanders et al., 2019). However, some meta-analysis showed that long hours of exercise per week tended to be associated with improved cognitive performance (Firth et al., 2017), and there is also evidence to support that structured, longer duration, and multimodal exercise intervention programs can better improve cognitive performance and overall function in older adults (Kirk-Sanchez and McGough, 2014). Longer exercises durations may result in greater cognitive benefits for older adults (Hewston et al., 2021). The reasons for these differences may be due to the large variation in the total weekly intervention duration, frequency, and intensity of the exercise program across the different studies included. Another possible reason for the results is that the number of included studies was small and the subgroup analysis was more confounded by random errors. Further studies are needed regarding the dose-effect of exercise prescription on cognitive function in elderly patients with T2DM. A Cochrane review and an RCT found that exercise had no benefit for cognitively healthy older adults (Sink et al., 2015; Young et al., 2015). However, we performed a subgroup analysis based on the presence or absence of cognitive impairment and found that exercise interventions were beneficial for both older patients with T2DM with and without cognitive impairment, with no significant differences. Previous systematic reviews have also supported the effectiveness of physical activity on cognitive performance in older adults with and without cognitive impairment (Northey et al., 2018; Falck et al., 2019), such as healthy individuals (Sáez de Asteasu et al., 2017), individuals with mild cognitive impairment (Song et al., 2018), and individuals with dementia (Groot et al., 2016; Jia et al., 2019). This suggests that exercise not only improves cognitive function (Ludyga et al., 2016; Falck et al., 2019), but also prevents (Bherer et al., 2013) and reduces cognitive decline (Musich et al., 2017).

This study has several limitations that might have affected its conclusions and significance. First, just five experiments with a modest number of samples were combined. Second, the methodological quality of the included studies was assessed as “very low” using the GRADE method. Third, because the included studies used different cognitive assessment scales, this might have led to the significant heterogeneity of the results. Despite these limitations, our findings still have some implications.

The current study’s main strength is that it provides an updated summary of RCTs of the effects of regular exercise on cognitive function in elderly patients with T2DM, which implies prospective observations and a causal rationale. Second, we excluded a large number of studies in which exercise was used as an adjunctive component of another intervention, such as cognitive training combined with exercise (Shellington et al., 2018) and diet combined with exercise training (Rapp et al., 2017; Espeland et al., 2018), and included only studies in which exercise was the only intervention. The findings of this research imply that the improvement of cognitive function by exercise in elderly patients with T2DM is practical and generalizable. To our knowledge, this is the first research to examine the effects of exercise on cognitive performance in older people with T2DM using a systematic review and meta-analysis. As a consequence, the findings of this research might be used as a recommendation for the implementation of exercise in the senior T2DM patient population.

Furthermore, only a few original articles have been published on the effects of physical exercise on specific domains of cognitive function such as executive function, attention, and memory in elderly patients with T2DM. Thus, further research is needed. Future research should expand intervention protocols and cognitive assessment areas, improve the research quality, and investigate the effects of various types of exercise modalities on physical inactivity and cognitive impairment in older adults with T2DM to develop a healthy lifestyle and exercise prescription recommendations to prevent the onset of dementia.

## Conclusion

The result of the systematic reviews and meta-analyses performed in this study suggest that both single-modal and multimodal exercise regularly for >3 months can enhance global cognitive function in older patients with T2DM, regardless of cognitive impairment. However, with the limited inclusion and considerable heterogeneity of the studies included, this finding should be treated with caution. To give further evidence, more RCTs implementing standardized study designs are required. Exercise’s impact on specific cognitive function domains in older patients with T2DM should also be investigated.

## Data Availability Statement

The original contributions presented in the study are included in the article/Supplementary Material, further inquiries can be directed to the corresponding author/s.

## Author Contributions

Y-HC and ZW conceived and designed the study, carried out the literature searches, extracted the data, and wrote the manuscript. Y-HC, ZW, and L-YF assessed the study quality and performed the statistical analysis. G-XN revised the manuscript. All authors contributed to the article and approved the submitted version.

## Conflict of Interest

The authors declare that the research was conducted in the absence of any commercial or financial relationships that could be construed as a potential conflict of interest.

## Publisher’s Note

All claims expressed in this article are solely those of the authors and do not necessarily represent those of their affiliated organizations, or those of the publisher, the editors and the reviewers. Any product that may be evaluated in this article, or claim that may be made by its manufacturer, is not guaranteed or endorsed by the publisher.
