# Supplementary material for: Effect of Exercise on the Cognitive Function of Older Patients With Type 2 Diabetes Mellitus: A Systematic Review and Meta-Analysis
Source: Front Hum Neurosci. 2022 Apr 28;16:876935. doi: 10.3389/fnhum.2022.876935 (PMC9096085; doi:10.3389/fnhum.2022.876935)
Supplement: Supplementary file 1 [file Data_Sheet_1.PDF]

Pubmed：276

((((("Diabetes Mellitus, Type 2"[Mesh]) OR "Type 2 Diabetes"[Title/Abstract]) OR "Diabetes Mellitus"[Title/Abstract]) OR NIDDM[Title/Abstract] OR MODY[Title/Abstract] OR T2DM[Title/Abstract] OR T2D[Title/Abstract] OR "non-insulin* depend*"[Title/Abstract] OR "noninsulin* depend*"[Title/Abstract] OR "non insulindepend*"[Title/Abstract] OR noninsulindepend*[Title/Abstract])) AND (("Exercise*"[Mesh]) OR ("muscle strength"[Title/Abstract] OR "muscle strengthening"[Title/Abstract] OR "muscle-strengthening"[Title/Abstract] OR "weight lifting"[Title/Abstract] OR weight-lifting[Title/Abstract] OR "weight bearing"[Title/Abstract] OR weight-bearing[Title/Abstract] OR "weight training"[Title/Abstract] OR "circuit training"[Title/Abstract] OR "strength exercise"[Title/Abstract] OR "strengthening exercise"[Title/Abstract] OR "strength training"[Title/Abstract] OR "resistance exercise"[Title/Abstract] OR "resistance training"[Title/Abstract] OR "progressive resistance"[Title/Abstract] OR "Physical Exercise"[Title/Abstract] OR "Isometric Exercise"[Title/Abstract] OR "aerobic exercise"[Title/Abstract] OR "aerobic training"[Title/Abstract] OR "exercise therapy"[Title/Abstract]))) AND ("cogniti*"[Title/Abstract] OR "executive"[Title/Abstract] OR "attention"[Title/Abstract] OR "memory"[Title/Abstract] OR "brain"[Title/Abstract] OR "neurocognitive"[Title/Abstract] OR "recollection"[Title/Abstract] OR "learning"[Title/Abstract] OR "problem solving"[Title/Abstract] OR "concentration"[Title/Abstract])) AND (“randomized controlled trial” or random* or placebo[Title/Abstract])

Web of Science：542

#5 #1 AND #2 AND #3 AND #4

#4 TS=(randomized controlled trial or random* or placebo)

#3 TS=("Exercise*" OR "muscle strength" OR "muscle strengthening" OR "muscle-strengthening" OR "weight lifting" OR weight-lifting OR "weight bearing" OR weight-bearing OR "weight training" OR "circuit training" OR "strength exercise" OR "strengthening exercise" OR "strength training" OR "resistance exercise" OR "resistance training" OR "progressive resistance" OR "Physical Exercise" OR "Isometric Exercise" OR "aerobic exercise" OR "aerobic training" OR "exercise therapy")

#2 TS=("Diabetes Mellitus, Type 2" OR "Type 2 Diabetes" OR "Diabetes Mellitus" OR NIDDM OR MODY OR T2DM OR T2D OR "non-insulin* depend*" OR "noninsulin* depend*" OR "non insulindepend*" OR noninsulindepend*)

#1 TS=(“cogniti*” OR “executive” OR “attention” OR “memory” OR “brain” OR “neurocognitive” OR “recollection” OR “learning” OR “problem solving” OR “concentration”)

Embase:767

#11 #5 AND #8 AND #9 AND #10

#10 #6 OR #7

#9 #3 OR #4

#8 #1 OR #2

#7 'diabetes mellitus, type 2':ab,ti OR 'type 2 diabetes':ab,ti OR 'diabetes mellitus':ab,ti OR 'niddm':ab,ti OR 'mody':ab,ti OR 't2dm':ab,ti OR 't2d':ab,ti OR 'non-insulin* depend*':ab,ti

OR 'noninsulin* depend*':ab,ti OR 'non insulindepend*':ab,ti OR 'noninsulindepend*':ab,ti
#6 'non insulin dependent diabetes mellitus'/exp
#5 'randomized controlled trial':ab,ti OR 'placebo':ab,ti OR 'random*':ab,ti OR 'rct':ab,ti
#4 'cogniti*':ab,ti OR 'executive':ab,ti OR 'attention':ab,ti OR 'memory':ab,ti OR 'brain':ab,ti OR 'neurocognitive':ab,ti OR 'recollection':ab,ti OR 'learning':ab,ti OR 'problem solving':ab,ti OR 'concentration':ab,ti
#3 'cognition'/exp
#2 'exercise*':ab,ti OR 'muscle strength':ab,ti OR 'muscle strengthening':ab,ti OR 'muscle-strengthening':ab,ti OR 'weight lifting':ab,ti OR 'weight-lifting':ab,ti OR 'weight bearing':ab,ti OR 'weight-bearing':ab,ti OR 'weight training':ab,ti OR 'circuit training':ab,ti OR 'strength exercise':ab,ti OR 'strengthening exercise':ab,ti OR 'strength training':ab,ti OR 'resistance exercise':ab,ti OR 'resistance training':ab,ti OR 'progressive resistance':ab,ti OR 'physical exercise':ab,ti OR 'isometric exercise':ab,ti OR 'aerobic exercise':ab,ti OR 'aerobic training':ab,ti OR 'exercise therapy':ab,ti
480,824
#1 'exercise'/exp

Ovid:265

1	(cogniti* or executive or attention or memory or brain or neurocognitive or recollection or learning or problem solving or concentration).ti,ab,kw.
2	(Diabetes Mellitus, Type 2 or type 2 diabetes or Diabetes Mellitus or NIDDM or MODY or T2DM or T2D or non-insulin* depend* or noninsulin* depend* or non insulindepend* or noninsulindepend*).ti,ab,kw.
3	(Exercise* or muscle strength or muscle strengthening or muscle-strengthening or weight lifting or weight-lifting or weight bearing or weight-bearing or weight training or circuit training or strength exercise or strengthening exercise or strength training or resistance exercise or resistance training or progressive resistance or Physical Exercise or Isometric Exercise or aerobic exercise or aerobic training or exercise therapy).ti,ab,kw.
4	(randomized controlled trial or random* or placebo).ti,ab,kw.
5	1 and 2 and 3 and 4

Cochrane:1249

ID Search
#1 MeSH descriptor: [Exercise] explode all trees
#2 (Exercise*):ab,ti,kw OR (muscle strength):ab,ti,kw OR (muscle strengthening):ab,ti,kw OR (muscle-strengthening):ab,ti,kw OR (weight lifting):ab,ti,kw OR (weight-lifting):ab,ti,kw OR (weight bearing):ab,ti,kw OR (weight-bearing):ab,ti,kw OR (weight training):ab,ti,kw OR (circuit training):ab,ti,kw OR (strength exercise):ab,ti,kw OR (strengthening exercise):ab,ti,kw OR (strength training):ab,ti,kw OR (resistance exercise):ab,ti,kw OR (resistance training):ab,ti,kw OR (progressive resistance):ab,ti,kw OR (Physical Exercise):ab,ti,kw OR (Isometric Exercise):ab,ti,kw OR (aerobic exercise):ab,ti,kw OR (aerobic training):ab,ti,kw OR (exercise therapy):ab,ti,kw

#3 MeSH descriptor: [Cognition] explode all trees
#4 (cogniti*):ab,ti,kw OR (executive):ab,ti,kw OR (attention):ab,ti,kw OR (memory):ab,ti,kw OR (brain):ab,ti,kw OR (neurocognitive):ab,ti,kw OR (recollection):ab,ti,kw OR (learning):ab,ti,kw OR (problem solving):ab,ti,kw OR (concentration):ab,ti,kw
#5 (Type 2 Diabetes):ab,ti,kw OR (Diabetes Mellitus):ab,ti,kw OR (NIDDM):ab,ti,kw OR (MODY):ab,ti,kw OR (T2DM):ab,ti,kw OR (T2D):ab,ti,kw OR (non-insulin* depend*):ab,ti,kw OR (noninsulin* depend*):ab,ti,kw OR (non insulindepend*):ab,ti,kw OR (noninsulindepend*):ab,ti,kw
#6 MeSH descriptor: [Diabetes Mellitus, Type 2] explode all trees
#7 #1 OR #2
#8 #3 OR #4
#9 #5 OR #6
#10 #7 AND #8 AND #9

Scopus:1587
1 (TITLE-ABS-KEY("cogniti*" OR "executive" OR "attention" OR "memory" OR "brain" OR "neurocognitive" OR "recollection" OR "learning" OR "problem solving" OR "concentration"）
2 (TITLE-ABS-KEY("Diabetes Mellitus, Type 2" OR "Type 2 diabetes" OR "Diabetes Mellitus" OR "NIDDM" OR "MODY" OR "T2DM" OR "T2D" OR "non-insulin* depend*" OR "noninsulin* depend*" OR "non insulindepend*" OR "noninsulindepend*"）
3 (TITLE-ABS-KEY("Exercise*" OR "muscle strength" OR "muscle strengthening" OR "muscle-strengthening" OR "weight lifting" OR "weight-lifting" OR "weight bearing" OR "weight-bearing" OR "weight training" OR "circuit training" OR "strength exercise" OR "strengthening exercise" OR "strength training" OR "resistance exercise" OR "resistance training" OR "progressive resistance" OR "Physical Exercise" OR "Isometric Exercise" OR "aerobic exercise" OR "aerobic training" OR "exercise therapy"）
4 (TITLE-ABS-KEY("randomized controlled trial" OR "placebo" OR "random*" OR "RCT")
1 AND 2 AND 3 AND 4

EBSCO 包括 Meadline: 248+5
#1 ("cogniti*" OR "executive" OR "attention" OR "memory" OR "brain" OR "neurocognitive" OR "recollection" OR "learning" OR "problem solving" OR "concentration")
#2 ("Diabetes Mellitus, Type 2" OR "Type 2 diabetes" OR "Diabetes Mellitus" OR "NIDDM" OR "MODY" OR "T2DM" OR "T2D" OR "non-insulin* depend*" OR "noninsulin* depend*" OR "non insulindepend*" OR "noninsulindepend*")
#3 ("Exercise*" OR "muscle strength" OR "muscle strengthening" OR "muscle-strengthening" OR "weight lifting" OR "weight-lifting" OR "weight bearing" OR "weight-bearing" OR "weight training" OR "circuit training" OR "strength exercise" OR "strengthening exercise" OR "strength training" OR "resistance exercise" OR "resistance training" OR "progressive resistance" OR "Physical Exercise" OR "Isometric Exercise" OR "aerobic exercise" OR "aerobic training" OR "exercise therapy")
#4 ("randomized controlled trial" OR "placebo" OR "random*" OR "RCT"))
(random*)

#5 #1 AND #2 AND #3 AND #4

Proquest：129

AB,TI("cogniti*" OR "executive" OR "attention" OR "memory" OR "brain" OR "neurocognitive" OR "recollection" OR "learning" OR "problem solving" OR "concentration") AND AB,TI("Diabetes Mellitus, Type 2" OR "Type 2 diabetes" OR "Diabetes Mellitus" OR "NIDDM" OR "MODY" OR "T2DM" OR "T2D" OR "non-insulin* depend*" OR "noninsulin* depend*" OR "non insulindepend*" OR "noninsulindepend*") AND AB,TI("Exercise*" OR "muscle strength" OR "muscle strengthening" OR "muscle-strengthening" OR "weight lifting" OR "weight-lifting" OR "weight bearing" OR "weight-bearing" OR "weight training" OR "circuit training" OR "strength exercise" OR "strengthening exercise" OR "strength training" OR "resistance exercise" OR "resistance training" OR "progressive resistance" OR "Physical Exercise" OR "Isometric Exercise" OR "aerobic exercise" OR "aerobic training" OR "exercise therapy") AND AB,TI("randomized controlled trial" OR "placebo" OR "random*" OR "RCT")
